# Storage-Battery Light

**Published:** 1884-03

**Authors:** 


### Storage Battery Light.

A Pullman dining car of the 5:40 express to Leeds has been lit by six Swan incandescent lamps, supplied with electricity from one primary battery of twelve cells, the dimensions of the battery being: Length, 4 feet; breadth, 8 inches; and depth, 8 inches. The battery is of zinc and carbon, with a new depolarizing arrangement, the details of which have not been made public. The lamps diffused a bright, warm, and perfectly steady light, which was at no moment affected by the oscillation of the carriage, and which made it not only possible, but perfectly easy, to read a newspaper or book printed in small type. The result of other preliminary trials of the system has been that several railway companies, including the great Eastern, the Southeastern, and the London and Southwestern, have shown a desire to adopt it. The light can be turned on or off at pleasure, and it can, therefore, be used in the day when a train is passing through a tunnel. The inventors of the battery express a belief that they will be able to supply private dwellings with electric light for less than the estimate lately put forward by the Edison Company and the Golcher Company. The battery which was used on Thursday week weighed under 150 lbs., and one capable of supplying eighteen lights for eighteen continuous hours would weigh about 3 cwt. The inventors of the system are Mr. G. C. V. Holmes and Mr. F. E. Burke.

